# Supplementary material for: Alternative oxidase‐mediated respiration prevents lethal mitochondrial cardiomyopathy
Source: EMBO Mol Med. 2018 Dec 10;11(1):e9456. doi: 10.15252/emmm.201809456 (PMC6328925; doi:10.15252/emmm.201809456)
Supplement: Supplementary file 1 — Appendix [file EMMM-11-e9456-s001.pdf]

Rajendran et al. Alternative oxidase-mediated respiration prevents lethal mitochondrial cardiomyopathy

Appendix material

Table of Contents

Appendix Figure S1. Kidney manifestations in GRAC and GROX mice at P200. 2

Appendix Figure S2. Transcriptomic changes related to oxidative metabolism at P150. 3

Appendix Table S1. Pathway enrichment analysis of the transcriptomics data (FC>1.5 and *p*<0.05) from heart, kidney and liver. 4

Appendix Table S2. Absolute concentrations (µM) of metabolites in heart and liver tissue at P150. 7

Appendix Table S3. List of primary and secondary antibodies used in this study. 10

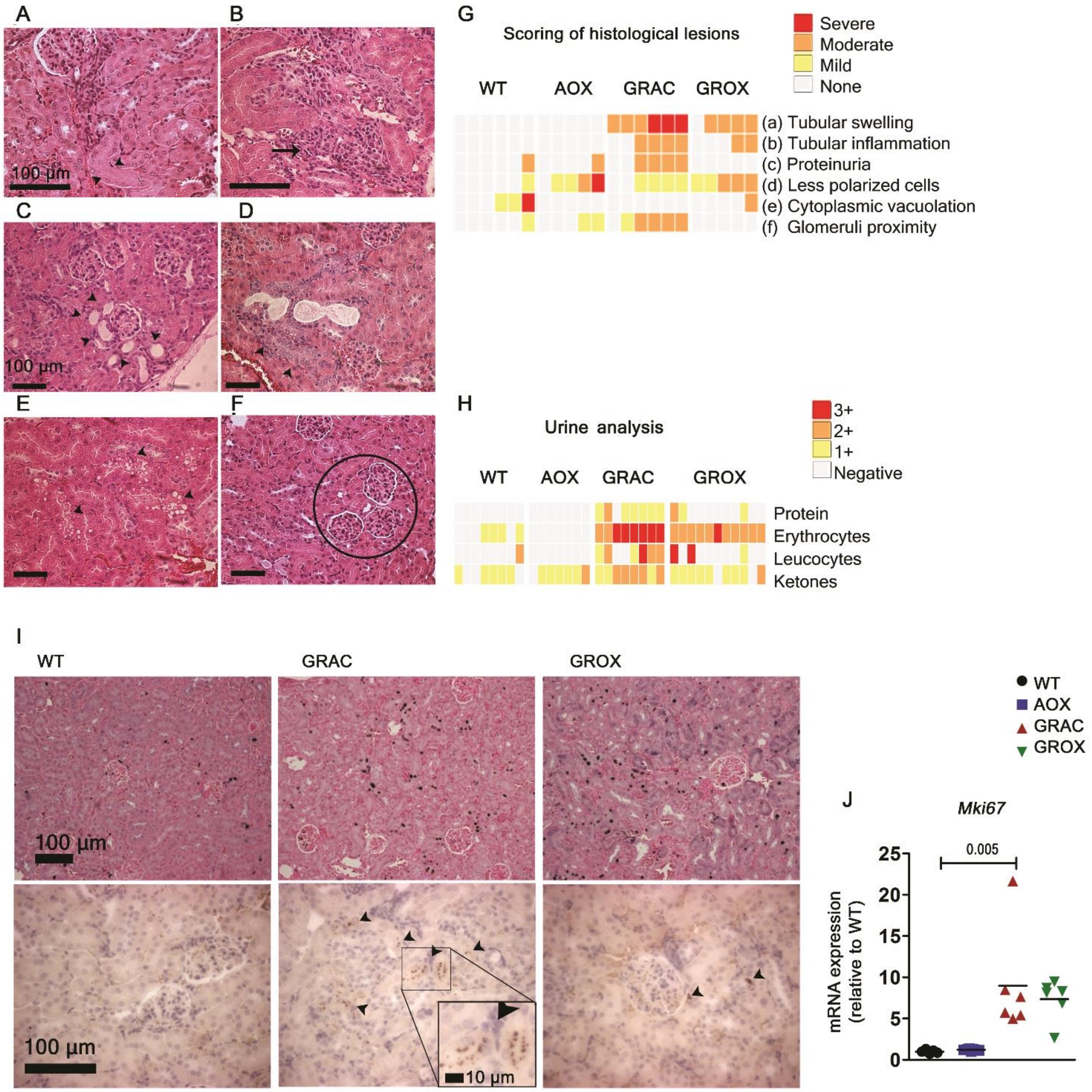

Appendix Figure S1. Kidney manifestations in GRAC and GROX mice at P200.

A-F. Distinct histological signs of tubular deterioration included (A) enlarged tubuli with diffuse nuclei (arrow heads), (B) inflammation (arrow), (C) proteinuria (arrow heads), (D) degenerating tubular cells (grey cells, arrow heads), (E) cytoplasmic vacuoles (arrow heads), and (F) glomerular proximity due to cortical atrophy (circled). Scale bar 100 µm.

G. Quantification of the histological findings.

H. Urine analysis.

I. Immunostaining for Ki-67 (proliferating cell marker) and caspase-3 (Apoptotic marker).

J. mRNA expression of *Ki-67* (*Mki67*) (One-way ANOVA with Tukey’s test).

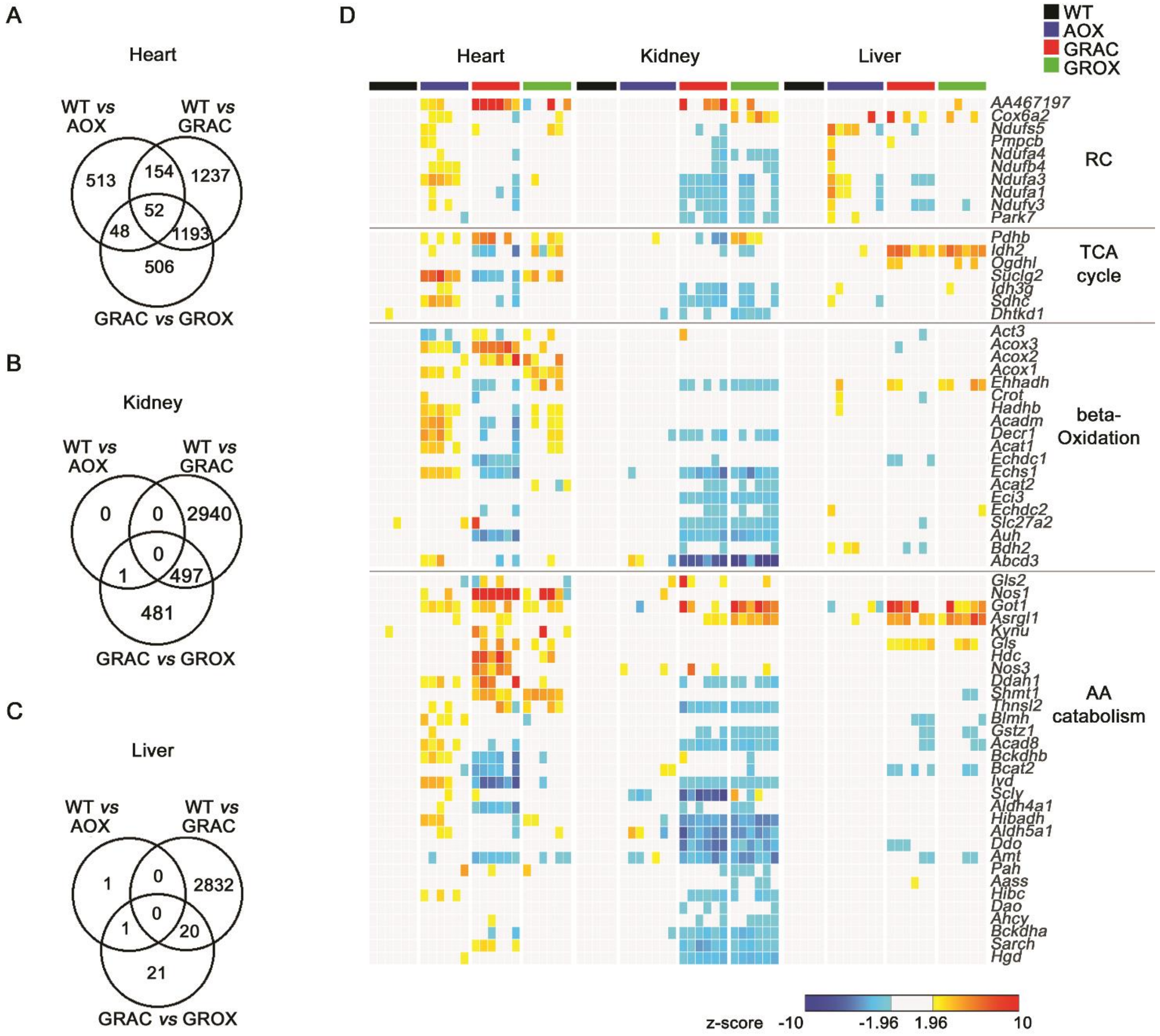

Appendix Figure S2. Transcriptomic changes related to oxidative metabolism at P150.

A-C. Venn diagrams showing the number of genes with significantly changed expression in the three comparisons (1: WT vs AOX, 2: WT vs GRAC, and 3: GRAC vs GROX) and their overlap in heart, kidney and liver.

D. AOX upregulated genes related to oxidative metabolism including RC, TCA cycle, beta-oxidation and amino acid (AA) catabolism in heart but not in liver or kidney. Heat map was generated from the z score values (WT as a reference population).

Appendix Table S1. Pathway enrichment analysis of the transcriptomics data (FC>1.5 and *p*<0.05) from heart, kidney and liver.

			WT vs GRAC comparison			GRAC vs GROX comparison		
**Tissue**	**Reactome ID**	**Pathway**	**Matches**	**p value**	**Regulation**	**Matches**	**p value**	**Regulation**
Heart	R-MMU-1474244	Extracellular matrix organization	65	2.0E-18	Up	74	1.5E-29	Down
Heart	R-MMU-1474290	Collagen formation	26	9.6E-11	Up	32	9.4E-19	Down
Heart	R-MMU-216083	Integrin cell surface interactions	25	2.8E-09	Up	29	5.0E-15	Down
Heart	R-MMU-9006934	Signaling by Receptor Tyrosine Kinases	57	1.9E-05	Up	65	1.6E-11	Down
Heart	R-MMU-186797	Signaling by PDGF	15	1.1E-04	Up	20	6.5E-10	Down
Heart	R-MMU-1566948	Elastic fibre formation	15	2.2E-07	Up	16	1.7E-09	Down
Heart	R-MMU-419037	NCAM1 interactions	8	6.5E-04	Up	12	2.6E-09	Down
Heart	R-MMU-3000157	Laminin interactions	9	2.2E-03	Up	13	6.4E-08	Down
Heart	R-MMU-109582	Hemostasis	74	3.3E-09	Up	60	1.4E-06	Down
Heart	R-MMU-2214320	Anchoring fibril formation	6	1.2E-02	Up	8	2.4E-05	Down
Heart	R-MMU-76002	Platelet activation, signaling and aggregation	42	1.7E-06	Up	34	9.6E-05	Down
Heart	R-MMU-8875878	MET promotes cell motility	12	3.2E-04	Up	11	3.8E-04	Down
Heart	R-MMU-114608	Platelet degranulation	24	1.8E-04	Up	20	1.6E-03	Down
Heart	R-MMU-76005	Response to elevated platelet cytosolic Ca2+	24	3.0E-04	Up	20	2.6E-03	Down
Heart	R-MMU-202733	Cell surface interactions at the vascular wall	22	6.5E-04	Up	-	-	-
Heart	R-MMU-1430728	Metabolism	99	1.2E-02	Down	94	4.2E-13	Up
Heart	R-MMU-70895	Branched-chain amino acid catabolism	7	1.3E-02	Down	6	7.3E-03	Up
Heart	R-MMU-71291	Metabolism of amino acids and derivatives	-	-	-	18	1.0E-02	Up
Heart	R-MMU-375165	NCAM signaling for neurite out-growth	-	-	-	12	2.4E-05	Down
Heart	R-MMU-422475	Axon guidance	-	-	-	35	3.5E-05	Down

			WT vs GRAC comparison			GRAC vs GROX comparison		
**Tissue**	**Reactome ID**	**Pathway**	**Matches**	**p value**	**Regulation**	**Matches**	**p value**	**Regulation**
Kidney	R-MMU-1640170	Cell Cycle	97	4.7E-18	Up	-	-	-
Kidney	R-MMU-194315	Signaling by Rho GTPases	56	2.3E-07	Up	-	-	-
Kidney	R-MMU-68886	M Phase	51	7.2E-07	Up	-	-	-
Kidney	R-MMU-6791312	TP53 Regulates Transcription of Cell Cycle Genes	13	2.4E-05	Up	-	-	-
Kidney	R-MMU-2555396	Mitotic Metaphase and Anaphase	33	3.0E-05	Up	-	-	-
Kidney	R-MMU-176187	Activation of ATR in response to replication stress	13	9.2E-05	Up	-	-	-
Kidney	R-MMU-73886	Chromosome Maintenance	15	2.6E-04	Up	-	-	-
Kidney	R-MMU-1430728	Metabolism	305	3.6E-59	Down	84	1.4E-04	Down
Kidney	R-MMU-196854	Metabolism of vitamins and cofactors	47	2.7E-13	Down	-	-	-
Kidney	R-MMU-71291	Metabolism of amino acids and derivatives	55	6.7E-13	Down	-	-	-
Kidney	R-MMU-556833	Metabolism of lipids	98	3.7E-12	Down	-	-	-
Kidney	R-MMU-211859	Biological oxidations	50	2.1E-10	Down	-	-	-
Kidney	R-MMU-70895	Branched-chain amino acid catabolism	13	1.7E-07	Down	-	-	-
Kidney	R-MMU-8957322	Metabolism of steroids	30	7.9E-07	Down	-	-	-
Kidney	R-MMU-390918	Peroxisomal lipid metabolism	14	8.1E-07	Down	-	-	-
Kidney	R-MMU-156580	Phase II - Conjugation of compounds	20	7.9E-05	Down	-	-	-
Kidney	R-MMU-211945	Phase I - Functionalization of compounds	27	6.0E-04	Down	-	-	-
Kidney	R-MMU-5419276	Mitochondrial translation termination	19	7.4E-04	Down	-	-	-
Kidney	R-MMU-5368287	Mitochondrial translation	19	8.4E-04	Down	-	-	-
Kidney	R-MMU-8963888	Chylomicron assembly	6	9.5E-04	Down	-	-	-
Kidney	R-MMU-174403	Glutathione synthesis and recycling	6	9.5E-04	Down	-	-	-

Tissue	Reactome ID	Pathway	WT vs GRAC comparison Matches	p value	Regulation	GRAC vs GROX comparison Matches	p value	Regulation
Liver	R-MMU-1640170	Cell Cycle	110	2.7E-17	Up	-	-	-
Liver	R-MMU-1474244	Extracellular matrix organization	69	1.1E-12	Up	-	-	-
Liver	R-MMU-194315	Signaling by Rho GTPases	73	9.4E-11	Up	-	-	-
Liver	R-MMU-2022090	Assembly of collagen fibrils and other multimeric structures	21	4.2E-09	Up	-	-	-
Liver	R-MMU-3000157	Laminin interactions	16	2.5E-08	Up	-	-	-
Liver	R-MMU-216083	Integrin cell surface interactions	25	1.3E-06	Up	-	-	-
Liver	R-MMU-419037	NCAM1 interactions	11	8.9E-06	Up	-	-	-
Liver	R-MMU-186797	Signaling by PDGF	18	6.6E-05	Up	-	-	-
Liver	R-MMU-176187	Activation of ATR in response to replication stress	14	9.4E-05	Up	-	-	-
Liver	R-MMU-9006934	Signaling by Receptor Tyrosine Kinases	68	4.9E-04	Up	-	-	-
Liver	R-MMU-1430728	Metabolism	188	9.2E-26	Down	-	-	-
Liver	R-MMU-556833	Metabolism of lipids	75	1.2E-10	Down	-	-	-
Liver	R-MMU-8957322	Metabolism of steroids	25	1.2E-06	Down	-	-	-
Liver	R-MMU-211859	Biological oxidations	35	1.5E-06	Down	-	-	-
Liver	R-MMU-166658	Complement cascade	18	1.8E-06	Down	-	-	-
Liver	R-MMU-174824	Plasma lipoprotein assembly, remodeling, and clearance	16	9.8E-06	Down	-	-	-
Liver	R-MMU-211945	Phase I - Functionalization of compounds	25	1.4E-05	Down	-	-	-
Liver	R-MMU-71291	Metabolism of amino acids and derivatives	32	3.3E-05	Down	-	-	-
Liver	R-MMU-381426	Regulation of Insulin-like Growth Factor (IGF) transport and uptake by Insulin-like Growth Factor Binding Proteins (IGFBPs)	23	9.5E-05	Down	-	-	-
Liver	R-MMU-196854	Metabolism of vitamins and cofactors	24	5.0E-04	Down	-	-	-

Selected pathways from pathway enrichment analysis of transcriptomics data from heart, kidney and liver at P150. Upregulated and downregulated genes (|FC|>1.5 and $p<0.05$) were analyzed separately. Benjamin-Hochberg correction was applied to *p* values.

Appendix Table S2. Absolute concentrations (μM) of metabolites in heart and liver tissue at P150.

	Heart						Liver					
	WT		GRAC		GROX		WT		GRAC		GROX	
**Metabolites**	**Mean**	**SD**	**Mean**	**SD**	**Mean**	**SD**	**Mean**	**SD**	**Mean**	**SD**	**Mean**	**SD**
2,3-Diphosphoglyceric acid	211.6	106.8	78.8	23.8	145.5	64.9	23.7	7.0	30.9	22.4	17.7	8.7
2-Hydroxyglutaric acid	20.0	5.4	45.6	25.3	17.6	1.6	19.3	4.1	17.3	2.0	12.3	5.7
2-Oxoglutaric acid	NA	NA	NA	NA	NA	NA	NA	NA	NA	NA	NA	NA
2-Oxoisovaleric acid	NA	NA	NA	NA	NA	NA	NA	NA	NA	NA	NA	NA
2-Phosphoglyceric acid	2.6	0.6	1.4	0.4	2.2	0.6	0.8	0.2	0.7	0.3	0.9	0.5
3-Phosphoglyceric acid	28.1	3.9	15.9	4.3	22.7	5.7	8.9	2.0	8.4	3.6	8.4	3.6
6-Phosphogluconic acid	5.8	0.5	5.8	0.6	6.0	0.6	43.3	11.4	22.7	10.6	33.2	5.3
Acetoacetyl CoA	NA	NA	0.1	NA	0.1	NA	3.7	2.4	2.0	1.3	2.5	1.7
Acetyl CoA	14.3	5.5	13.5	4.0	15.5	3.8	4.7	1.2	1.2	0.5	1.9	0.9
Adenine	6.2	3.4	3.7	4.6	7.7	4.9	2.3	0.8	3.2	1.3	2.8	1.3
Adenosine	136.1	98.7	117.6	171.6	207.5	172.0	163.5	17.5	154.3	26.1	108.3	19.5
Adenylosuccinic acid	12.0	7.4	8.2	3.3	9.3	7.4	8.2	5.7	41.4	15.2	16.1	10.4
ADP	1322.8	312.2	986.2	296.6	1291.8	198.7	588.9	220.0	744.4	209.6	787.4	161.7
ADP-ribose	50.0	18.2	42.3	13.9	32.5	14.5	193.9	38.2	135.8	44.5	166.0	32.7
Ala	3086.7	527.0	3576.7	702.0	3148.7	290.6	3945.1	1656.8	3322.4	1109.5	5012.9	2126.9
AMP	1093.3	470.1	841.5	373.8	859.9	306.2	1671.4	134.1	1680.1	289.7	1670.7	215.4
Arg	169.3	11.8	166.3	45.3	168.1	27.8	5.3	0.1	7.6	3.5	10.9	4.4
Argininosuccinic acid	6.8	1.4	8.1	2.8	7.3	2.5	6.1	2.1	11.2	3.7	11.7	4.6
Asn	127.8	20.4	159.0	104.2	233.0	24.9	132.9	17.2	141.3	30.2	156.1	38.5
Asp	1178.9	366.6	667.1	286.1	1173.2	245.1	467.1	117.5	716.0	264.9	648.4	199.9
ATP	1503.7	443.8	1297.2	768.3	1701.0	172.3	187.1	158.9	234.3	108.0	334.4	154.4
Betaine	NA	NA	34.4	25.9	56.3	34.0	850.3	383.6	1721.4	662.9	1916.7	335.4
Betaine aldehyde	NA	NA	NA	NA	NA	NA	1.7	0.8	4.8	1.3	8.0	5.0
cAMP	0.6	0.1	0.7	0.2	0.7	0.2	0.5	0.1	1.2	0.7	1.2	0.7
Carbamoylphosphate	NA	NA	NA	NA	NA	NA	NA	NA	NA	NA	NA	NA
Carnitine	311.3	80.4	257.7	110.7	438.4	73.8	190.1	52.8	169.1	66.9	203.1	46.1
Carnosine	17.1	13.9	150.6	79.7	22.7	12.2	1.7	0.4	1.8	1.0	1.3	0.2
cGMP	0.0	0.0	0.0	0.0	0.0	0.0	0.0	NA	0.0	NA	NA	NA
Choline	87.8	17.8	48.8	32.3	52.6	26.3	117.1	16.8	79.7	51.7	104.0	27.9
cis-Aconitic acid	4.1	1.4	10.3	4.8	5.1	2.3	1.1	0.3	6.6	4.2	2.4	1.4
Citric acid	287.2	43.1	429.2	178.1	314.6	115.2	49.2	9.3	219.6	117.3	90.6	34.3
Citrulline	175.1	20.6	241.9	67.2	126.8	28.4	30.4	22.0	40.8	5.7	34.3	23.9
CoA	29.5	9.3	29.7	6.3	34.8	14.3	60.0	7.8	47.2	9.2	59.1	14.6
Creatine	9826.1	1921.4	10347.9	186.6	10580.1	1327.8	217.7	55.1	348.5	148.9	298.8	50.4
Creatinine	9.0	1.2	10.3	0.7	9.0	2.2	6.3	0.9	6.8	1.5	4.9	1.5
Cys	NA	NA	NA	NA	NA	NA	18.6	12.4	5.6	3.5	14.1	6.9
Cystathionine	NA	NA	NA	NA	0.4	NA	13.3	8.1	12.2	2.9	28.3	10.2
Dihydroxyacetone phosphate	186.4	48.1	139.0	67.3	178.0	39.9	62.4	18.0	43.3	13.5	103.2	28.3
Erythrose 4-phosphate	NA	NA	NA	NA	NA	NA	NA	NA	NA	NA	NA	NA
Folic acid	NA	NA	NA	NA	NA	NA	0.0	0.0	0.0	0.0	0.0	0.0
Fructose 1,6-diphosphate	106.1	49.3	123.4	72.7	127.1	87.3	5.8	1.3	6.7	2.9	22.3	8.1
Fructose 1-phosphate	4.1	2.6	5.3	4.5	9.1	7.4	16.9	8.0	6.5	2.7	22.3	11.6
Fructose 6-phosphate	69.2	14.9	57.9	27.6	94.0	28.0	77.6	26.5	22.6	16.4	51.6	22.1
Fumaric acid	291.9	64.4	274.7	101.8	329.5	194.8	85.1	46.7	440.6	229.6	262.5	143.4
Galactose 1-phosphate	7.2	1.2	5.7	1.4	8.1	1.5	39.7	23.8	7.3	3.8	15.8	5.1

GDP	47.7	8.4	31.0	8.0	40.4	9.9	27.6	14.1	21.2	5.1	28.8	10.7
Gln	6121.1	800.7	6283.1	534.2	4336.3	827.6	3811.9	545.4	3020.7	1042.6	2958.2	378.0
Glu	4110.9	1165.9	1893.7	586.0	4338.2	854.0	1115.1	113.6	2639.1	557.6	1658.1	196.4
Glucose 1-phosphate	12.8	3.0	14.1	3.3	18.0	6.1	19.6	9.5	4.2	2.7	9.3	4.5
Glucose 6-phosphate	223.8	49.5	193.0	78.1	315.4	98.4	278.1	106.7	86.8	65.1	185.9	80.1
Glutathione (GSH)	137.0	25.9	70.8	45.4	254.2	133.4	3006.2	1672.1	1582.8	1147.1	3660.7	1036.0
Glutathione (GSSG)	491.3	40.4	452.2	69.2	532.1	48.4	1875.3	354.0	2395.2	150.1	2432.0	695.8
Gly	511.1	47.1	637.4	154.8	879.8	77.2	1345.5	168.8	2286.1	471.2	1711.8	387.1
Glyceraldehyde 3-phosphate	3.2	2.1	3.6	1.3	7.3	7.5	2.6	2.0	2.5	1.4	7.8	3.6
Glycerol 3-phosphate	988.2	164.5	985.7	317.3	867.3	241.8	3243.0	365.9	1912.2	482.9	2505.7	498.0
Glycolic acid	10.7	6.1	2.4	NA	6.7	NA	26.0	NA	2.0	NA	3.0	2.9
Glyoxylate	NA	NA	NA	NA	NA	NA	NA	NA	NA	NA	NA	NA
GMP	38.4	13.3	27.1	10.6	34.8	6.5	300.9	39.6	270.1	41.8	267.1	36.4
GTP	67.6	13.4	56.1	28.6	73.5	16.4	17.1	13.6	13.7	4.2	22.6	9.1
Guanine	NA	NA	NA	NA	NA	NA	NA	NA	NA	NA	NA	NA
Guanosine	3.6	2.0	4.9	7.3	5.6	4.9	43.0	4.8	32.8	7.7	28.0	6.6
His	176.7	52.1	214.1	15.8	157.9	34.3	439.0	28.1	583.4	130.0	620.1	86.9
HMG CoA	0.5	0.0	0.5	0.0	0.5	0.1	0.5	0.0	0.5	0.1	0.4	NA
Homocysteine	NA	NA	NA	NA	NA	NA	NA	NA	NA	NA	NA	NA
Homoserine	2.8	NA	1.4	0.3	1.5	0.4	2.2	0.9	2.0	0.7	3.2	0.8
Hydroxyproline	19.8	12.7	25.7	18.1	22.1	4.8	21.8	8.0	24.5	11.4	18.4	4.4
Hypoxanthine	44.7	31.8	41.6	74.2	36.7	26.1	32.4	19.3	73.1	89.6	46.1	26.7
Ile	59.2	3.5	110.2	47.1	65.5	32.0	187.1	25.4	211.1	63.9	197.9	83.3
IMP	41.6	11.0	40.6	23.4	58.8	41.1	196.1	112.5	117.8	29.4	133.0	71.3
Inosine	122.2	70.6	95.1	160.2	144.9	106.8	210.6	56.9	148.5	36.3	202.1	96.7
Isocitric acid	NA	NA	NA	NA	NA	NA	NA	NA	NA	NA	NA	NA
Lactic acid	15596.9	3334.7	15570.5	4744.1	17045.4	5070.6	14077.6	3159.0	11091.2	1770.4	16531.4	4827.8
Leu	119.8	14.7	183.7	63.6	166.7	26.3	346.7	41.2	396.1	106.7	407.1	104.3
Lys	429.5	26.7	381.2	89.7	409.4	90.3	845.5	130.4	564.2	66.0	737.5	93.6
Malic acid	704.1	160.5	770.2	281.2	785.7	390.4	254.2	181.9	1507.4	774.8	860.0	469.7
Malonyl CoA	0.3	0.0	0.3	0.0	0.3	0.0	NA	NA	NA	NA	NA	NA
Met	56.5	8.4	74.3	12.2	116.2	21.1	77.1	9.4	73.4	22.7	95.3	12.8
Mevalonic acid	NA	NA	NA	NA	NA	NA	NA	NA	NA	NA	NA	NA
N,N-dimethylglycine	NA	NA	3.0	2.6	0.2	0.0	33.3	6.8	34.3	20.5	34.1	11.1
N-acetylglutamic acid	1.3	NA	1.6	0.2	2.7	0.5	47.0	20.2	70.9	18.2	71.7	43.6
NAD+	625.9	132.4	643.2	32.7	667.8	78.2	654.3	103.1	464.9	113.1	571.2	56.9
NADH	20.5	11.3	24.0	8.5	20.8	11.6	8.3	1.3	11.9	0.8	9.6	1.2
NADP+	28.2	10.7	30.1	3.9	32.1	7.0	24.0	6.6	21.2	9.9	27.0	8.8
NADPH	4.9	0.9	5.6	2.2	5.0	1.1	4.1	0.4	4.3	0.8	4.6	0.8
N-carbamoylaspartic acid	0.2	0.0	1.1	0.6	0.5	0.3	0.2	0.2	2.5	2.2	2.9	2.9
Ornithine	15.8	6.4	15.1	4.6	25.9	2.5	241.9	65.2	231.2	58.6	294.3	83.5
Phe	76.0	10.1	106.9	9.6	103.4	10.2	151.8	20.1	155.5	19.7	171.1	24.3
Phosphocreatine	8.5	4.5	9.4	5.8	10.4	2.8	3.9	1.3	2.5	2.0	3.5	1.6
Phosphoenolpyruvic acid	5.4	2.7	1.5	1.5	3.0	2.3	0.5	0.2	0.9	0.4	0.6	NA
Pro	NA	NA	210.6	112.2	84.2	73.8	275.0	116.9	306.9	141.9	284.5	84.3
PRPP	2.0	0.7	7.4	7.3	2.3	1.1	0.8	0.2	0.9	0.4	1.1	0.6
Putrescine	1.2	0.3	8.0	3.7	4.2	1.4	2.3	1.1	21.6	14.4	5.7	3.5
Pyruvic acid	33.0	17.0	21.4	5.3	33.7	11.4	106.1	30.0	105.5	40.2	134.2	18.4
Ribose 1-phosphate	31.4	7.8	36.1	27.4	46.9	24.1	147.7	24.6	89.0	40.2	106.5	32.6
Ribose 5-phosphate	7.5	2.3	5.7	1.3	6.0	1.5	22.5	6.6	22.9	6.0	22.2	3.2
Ribulose 5-phosphate	5.2	1.7	5.1	2.2	4.7	1.1	4.7	1.4	4.3	0.6	6.9	2.3

S-adenosylhomocysteine	0.8	0.0	0.9	NA	0.8	0.2	37.7	12.8	27.8	9.2	37.8	16.9
S-adenosylmethionine	20.5	1.3	21.9	2.9	21.2	2.4	46.4	9.4	45.2	20.5	65.9	3.1
Sarcosine	NA	NA	NA	NA	NA	NA	30.1	20.4	48.0	33.6	46.6	31.6
Sedoheptulose 7-phosphate	NA	NA	NA	NA	NA	NA	140.1	59.7	78.4	56.9	94.3	40.2
Ser	282.1	50.5	469.9	238.0	496.3	104.7	213.8	41.4	572.7	240.7	479.0	178.3
Spermidine	28.4	7.0	37.1	11.1	35.5	10.0	49.7	11.6	50.7	14.3	42.2	4.6
Spermine	38.7	15.6	26.9	6.2	35.1	9.6	7.7	2.1	6.1	2.6	5.8	1.5
Succinic acid	1071.1	280.0	731.5	414.0	722.8	405.0	828.9	400.0	946.0	336.0	946.9	254.2
Thr	319.2	25.5	429.3	56.6	493.3	28.9	299.6	62.7	535.5	150.0	522.1	112.2
Trp	37.5	12.4	41.6	19.0	37.7	11.1	54.7	10.2	50.7	13.1	54.9	13.3
Tyr	71.0	17.7	95.4	50.6	107.4	11.0	113.8	23.2	121.1	43.8	141.7	31.6
UDP-glucose	24.6	6.1	24.1	7.2	26.5	4.8	150.0	94.7	223.8	97.2	207.3	62.7
Urea	5689.4	1276.8	6674.8	941.2	6341.3	1544.4	5128.6	1171.5	6283.3	757.9	6098.6	1573.9
Uric acid	43.1	30.4	26.2	12.4	28.9	14.0	22.0	17.6	21.5	19.1	21.5	11.5
Val	118.8	17.6	178.6	47.9	137.5	37.5	359.1	54.2	388.4	106.8	367.0	144.2
Xanthine	20.3	15.3	18.4	28.2	17.7	10.4	42.3	28.8	72.6	89.1	57.3	37.9
XMP	0.1	0.0	0.1	0.0	0.1	0.1	0.1	0.0	0.2	0.2	0.0	0.0
Xylulose 5-phosphate	NA	NA	3.6	NA	NA	NA	3.7	2.8	2.4	NA	4.2	NA
β-Ala	3.9	4.1	7.4	4.5	6.6	3.1	227.4	149.6	311.8	202.0	255.3	90.6
γ-aminobutyric acid	15.4	3.3	7.7	9.2	13.7	12.2	25.0	5.4	35.1	16.2	38.4	15.6

Quantitation of 116 metabolites by capillary electrophoresis-mass spectrometry (CE-TOMFS and CE-QqQMS) in heart and liver tissue at P150. NA = not available.

Appendix Table S3. List of primary and secondary antibodies used in this study.

Experiment	Antibody	Catalog	Species	Vendor
IHC	glial fibrillary acidic protein (GFAP)	Z0334	rabbit	DAKO
IHC	4-hydroxynonenal	ab46545	rabbit	Abcam
IHC	Ki67	ab16667	rabbit	Abcam
WB	total OXPHOS antibody cocktail	MS603	mouse	Abcam
BNGE/WB	UQCRFS1 (cIII subunit RISP)	ab14746	mouse	Abcam
WB	voltage-gated ion channel 1 (VDACV1)	ab154856	rabbit	Abcam
WB	alternative oxidase (AOX)	custom-made	rabbit	21st Century Biochemicals
WB	NDUFA9 (cI subunit)	ab14713	mouse	Abcam
WB	SDHB (cII subunit)	ab14714	mouse	Abcam
BNGE/WB	ubiquinol cytochrome C reductase core protein I	ab110252	mouse	Abcam
WB	MT-CO1 (cIV subunit)	a14705	mouse	Abcam
WB	ATP synthase F1 subunit alpha (ATP5A)	ab14748	mouse	Abcam
BNGE/WB	anti-rabbit IgG, HRP-linked	7074	goat	Cell Signaling Technologies
BNGE/WB	anti-mouse IgG, HRP-linked	7076	goat	Cell Signaling Technologies
WB	dinitrophenol	ab178020	rabbit	Abcam
WB	nitric oxide synthase 1 (NOS1)	sc-5302	mouse	Santa Cruz
WB	nitrotyrosine	9691	mouse	Cell Signaling Technologies
WB	phospho-phospholamban (Ser16/Thr17)	8496	mouse	Cell Signaling Technologies
WB	phospholamban	14562	rabbit	Cell Signaling Technologies
BNGE	electron transfer flavoprotein subunit alpha (ETFA)	ab110316	mouse	Abcam

UQCRFS1: ubiquinol-cytochrome C reductase, Rieske iron-sulfur polypeptide 1; NDUFA9: NADH:ubiquinone oxidoreductase subunit A9; SDHB: succinate dehydrogenase complex iron-sulfur subunit B; MT-CO1: mitochondrially-encoded cytochrome C oxidase I; IgG: immunoglobulin G
