# Supplementary material for: Alternative oxidase‐mediated respiration prevents lethal mitochondrial cardiomyopathy
Source: EMBO Mol Med. 2018 Dec 10;11(1):e9456. doi: 10.15252/emmm.201809456 (PMC6328925; doi:10.15252/emmm.201809456)
Supplement: Supplementary file 2 — Expanded View Figures PDF [file EMMM-11-e9456-s002.pdf]

Expanded View Figures

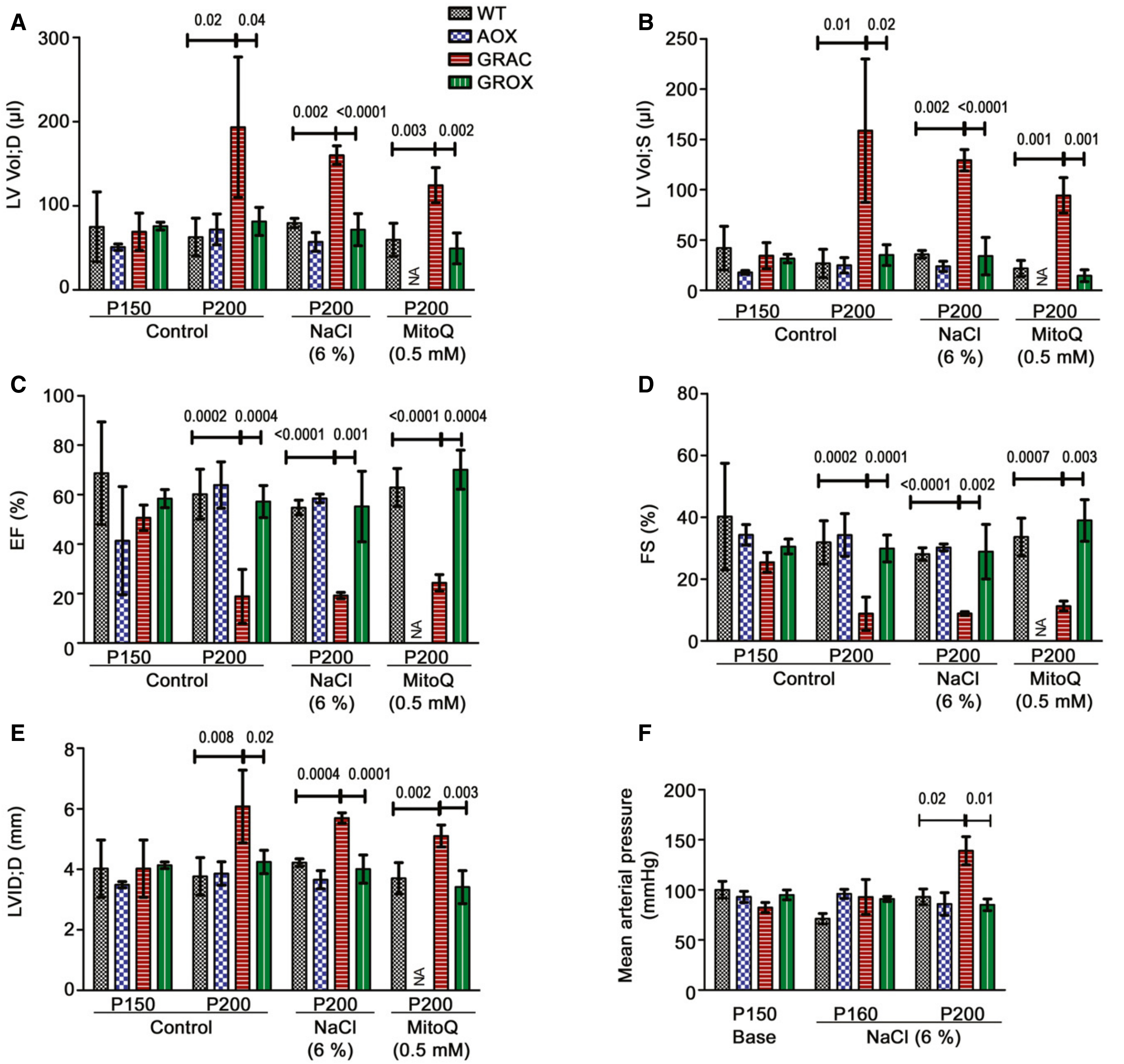

Figure EV1. Salt and antioxidant feeding have no effect on cardiac functions at P200.

A–E Echocardiography data from mice fed standard or 6% NaCl-supplemented chow or drinking water with 0.5 mM mitoQ (n = 4–6/group). Left ventricle volume at (A) diastolic (LV Vol;D) and (B) systolic (LV Vol;S) states, (C) ejection fraction (EF), (D) fractional shortening (FS), and (E) left ventricle internal dimensions (LVID; d-D).

F Mean arterial blood pressure in the salt-fed GRAC mice at baseline (P150), at P160, and at end stage (P200) (n = 4–6/group).

Data information: Bar graphs represent mean ± SD. One-way ANOVA followed by unpaired t-test with Welch's correction.

 © 2018 The Authors

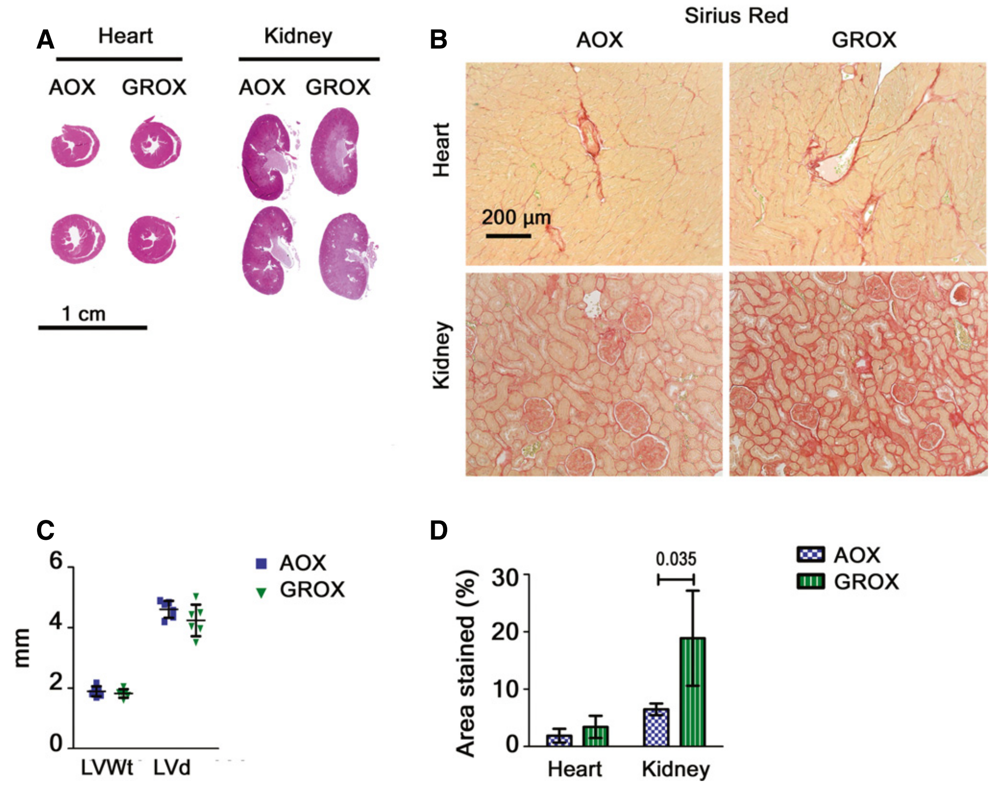

Figure EV2. AOX provides permanent protection from cardiomyopathy and kidney atrophy.

A Cross-sections of heart and kidney from old (P600) AOX and GROX mice.
B Heart and kidney fibrosis.
C Left ventricle wall thickness (LVWt) and diameter (LVd) measured from heart cross-sections (*n* = 6/group).
D Quantification of Sirius Red staining (*n* = 6–7/group, Mann–Whitney *U*-test).

Data information: Scatter plot (C) and bar graph (D) represent mean ± SD.

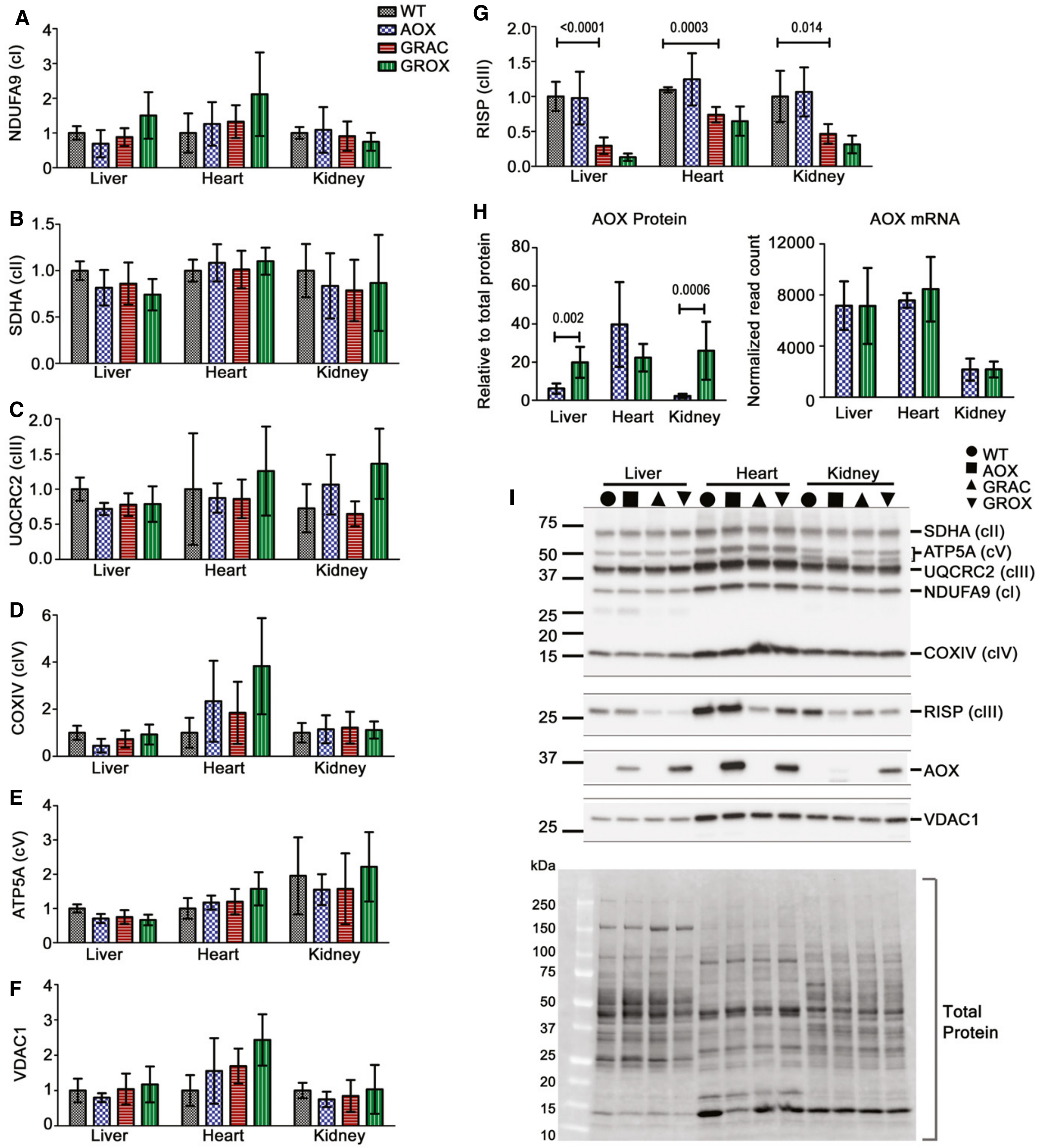

Figure EV3. AOX does not affect mitochondrial mass in the affected tissues.

A–G Quantification (*n* = 5–6/group) of mitochondrial respiratory chain subunits by Western blotting with antibodies against (A) NDUFA9 (cI), (B) SDHA (cII), (C) UQCRC2 subunit (cIII), (D) COXIV (cIV), (E) ATP5A (cV), (F) VDAC1, and (G) RISP (cIII).

H Protein (*n* = 6/group) and mRNA expression of AOX (*n* = 6/group).

I A representative Western blot with one sample per group and total protein visualization.

Data information: Bar graphs represent mean ± SD. One-way ANOVA followed by unpaired *t*-test with Welch's correction for selected comparisons (graph G), and Mann–Whitney's *U*-test for planned comparison (graph H). Number of technical replicates per sample was one (A–H).
Source data are available online for this figure.

 © 2018 The Authors

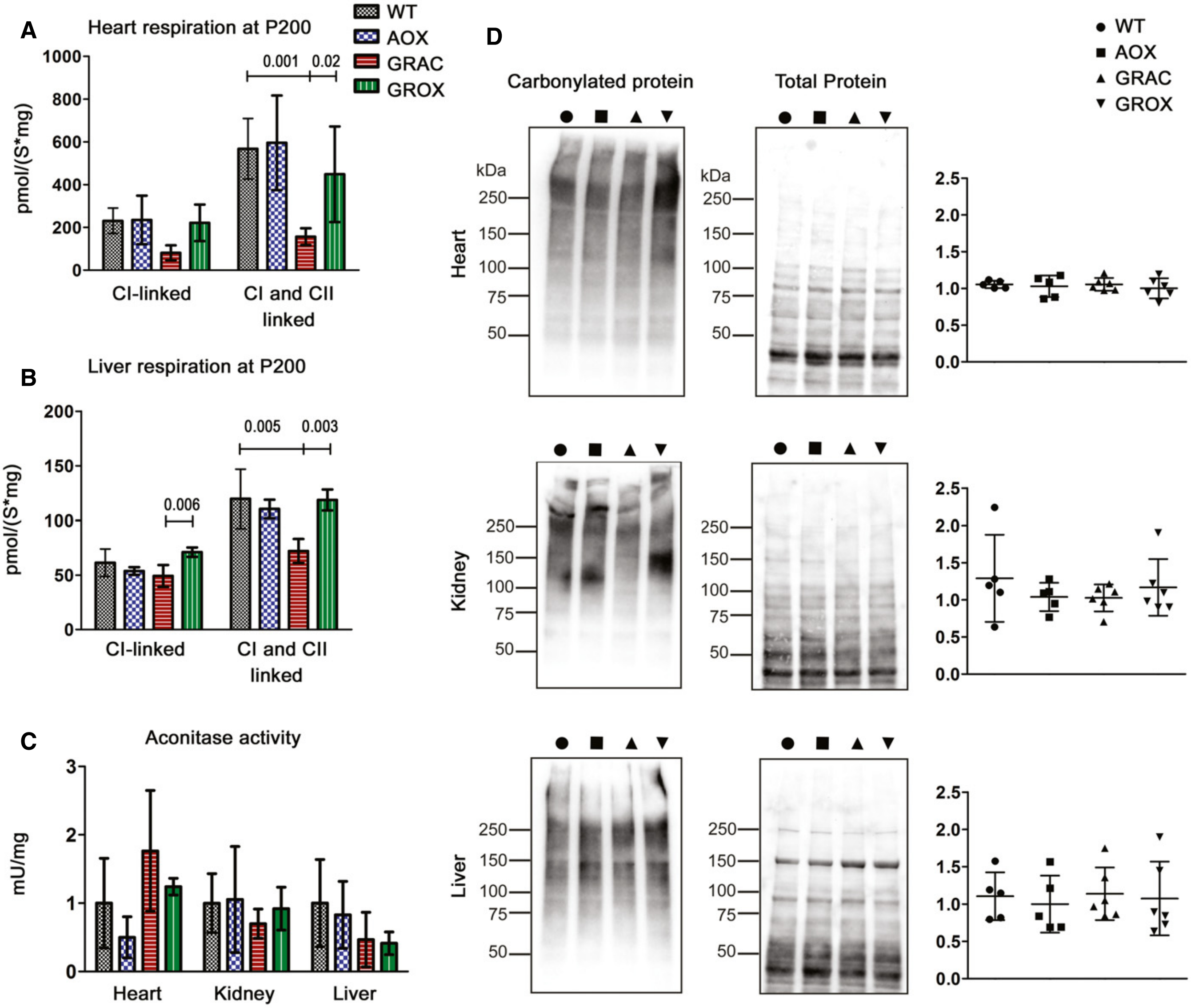

Figure EV4. Respiration and ROS damage markers in heart, kidney, and liver tissue at P200.

A, B CI-linked, and cI- and cII-linked respiration in (A) heart and (B) liver homogenates at P200 (n = 4–6/group).

C Aconitase activity, a marker of ROS damage, in mitochondrial fractions from frozen tissues (n = 4/group).

D Analysis of carbonylated proteins in total tissue lysates using on-filter labeling with 2,4-dinitrophenylhydrazine and detection with anti-dinitrophenol antibody. Representative Western blots and quantification are shown.

Data information: Bar graphs represent mean ± SD. One-way ANOVA followed by unpaired t-test with Welch's correction was used (for graphs A and B).
