# Supplementary figures and images for: Alternative oxidase‐mediated respiration prevents lethal mitochondrial cardiomyopathy
Source: EMBO Mol Med. 2018 Dec 10;11(1):e9456. doi: 10.15252/emmm.201809456 (PMC6328925; doi:10.15252/emmm.201809456)

Fig EV3I. Representative Western blots

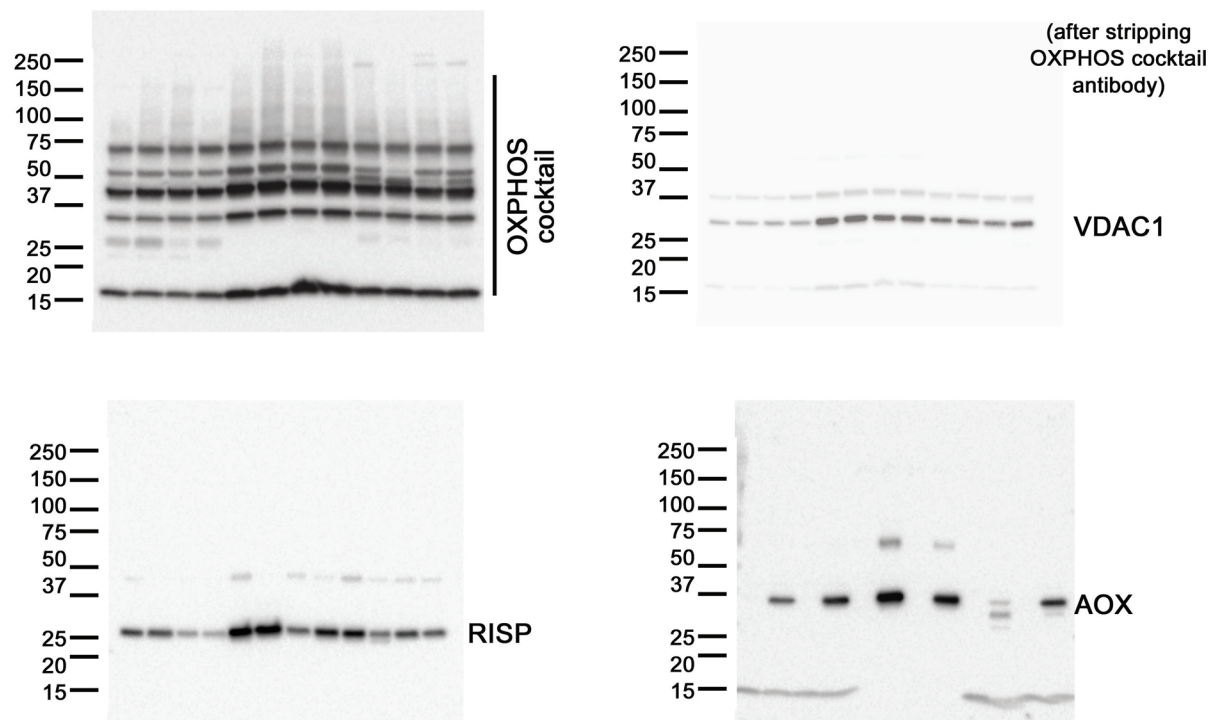

Supplement: Supplementary file 3 — Source Data for Expanded View [file EMMM-11-e9456-s005.pdf]

Fig 6A. Blue native PAGE

Heart

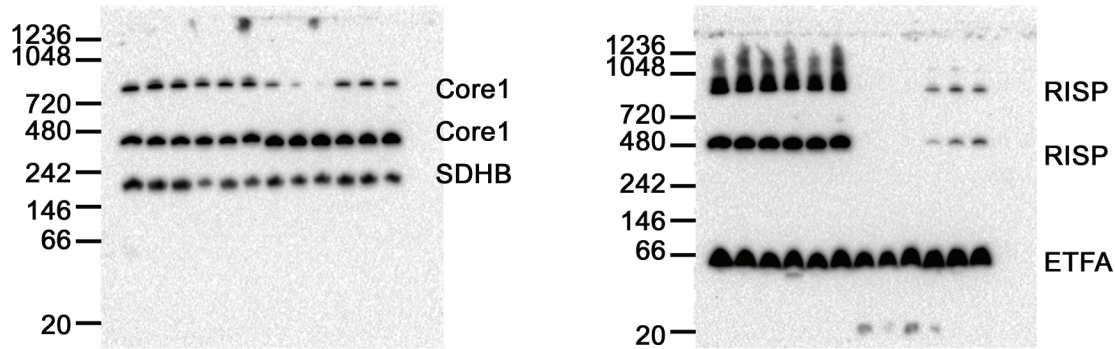

Kidney

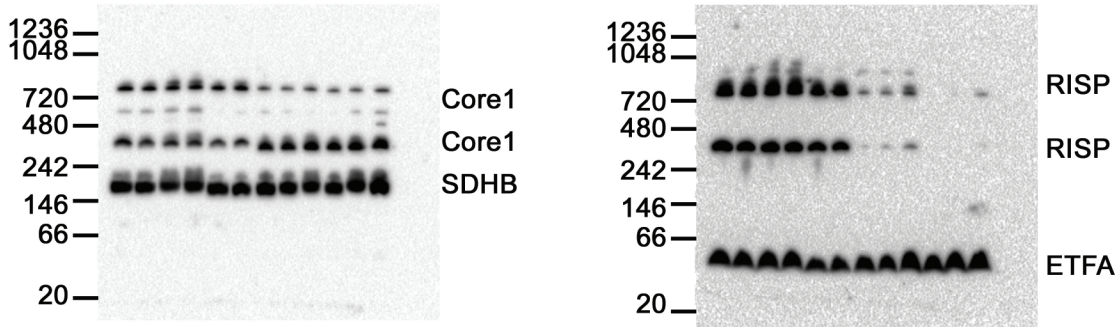

Liver

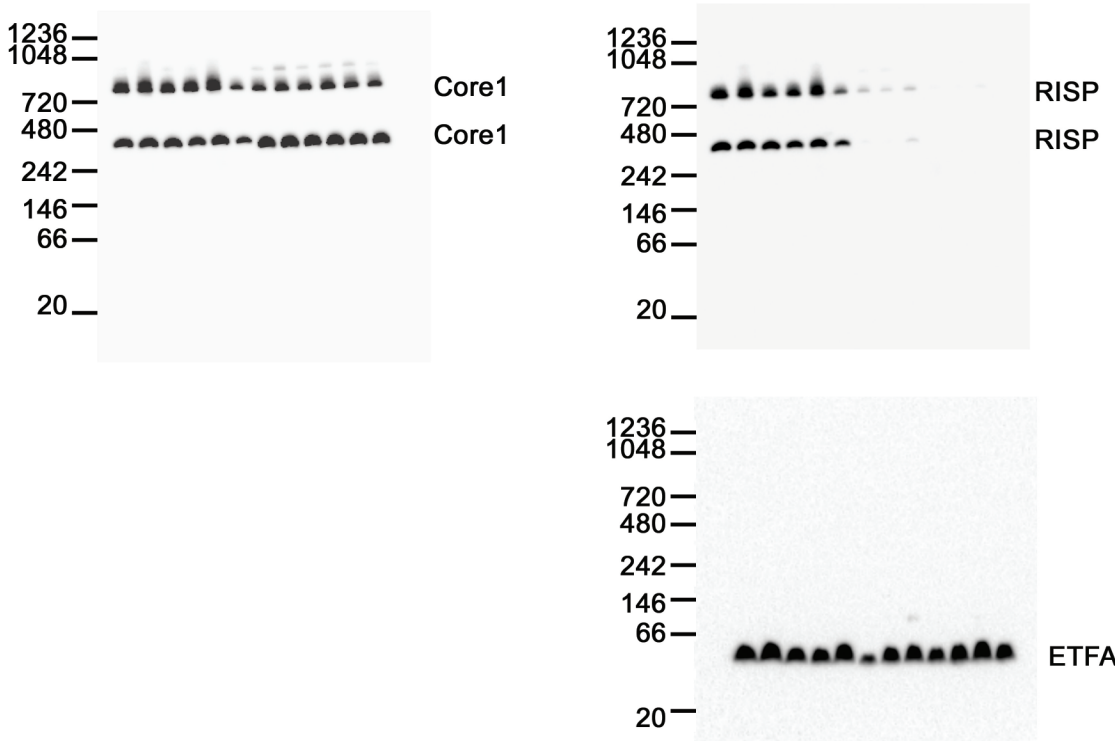

Supplement: Supplementary file 5 — Source Data for Figure 6 [file EMMM-11-e9456-s003.pdf]

Fig 8G. NOS1 antibody

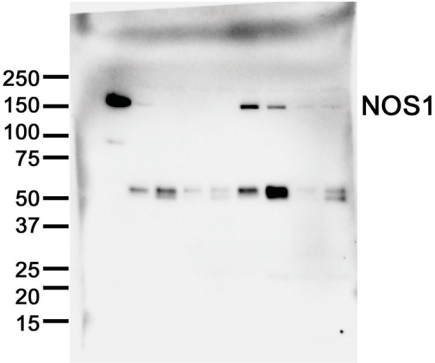

Supplement: Supplementary file 6 — Source Data for Figure 8 [file EMMM-11-e9456-s004.pdf]
